# Morphologic, Immunohistochemical, and Molecular Features of Laser-Ablated Thyroid Nodules: Diagnostic Pitfalls and Differential Diagnosis with Thyroid Carcinoma

**DOI:** 10.3390/ijms27135880

**Published:** 2026-06-30

**Authors:** Pietro Tralongo, Fernanda Russotto, Valeria Zuccalà, Vincenzo Fiorentino, Marina Gloria Micali, Mariausilia Franchina, Ludovica Pepe, Walter Giordano, Gabriele Ricciardi, Mariagiovanna Ballato, Emanuela Germanà, Emilia Magliolo, Serenella Ristagno, Esther Diana Rossi, Maurizio Martini, Guido Fadda

**Affiliations:** 1 Department of Biomedical, Dental and Morphological and Functional Imaging Sciences, University of Messina, 98125 Messina, Italy; waltergiordano1997g@gmail.com (W.G.); gricciardi1998@gmail.com (G.R.); mariagiovannaballato96@gmail.com (M.B.); emanuelagermana@hotmail.it (E.G.); 2 Department of Human Pathology of the Adulthood and of the Developing Age “Gaetano Barresi”, University of Messina, 98125 Messina, Italy; russottofernanda@gmail.com (F.R.); valeria.zuccala@unime.it (V.Z.); vincenzo.fiorentino@unime.it (V.F.); micalimarina@yahoo.it (M.G.M.); mariausilia.franchina@asp.messina.it (M.F.); ludopepe97@gmail.com (L.P.); guido.fadda@unime.it (G.F.); 3 Department of Pathology, “San Vincenzo” Hospital, 98039 Taormina, Italy; emilia.magliolo@asp.messina.it; 4 Department of Oncology, Section of Endocrine Surgery, “San Vincenzo” Hospital, 98039 Taormina, Italy; serenellaristagno@gmail.com; 5 Division of Anatomic Pathology and Histology, Catholic University of Sacred Heart, Fondazione Policlinico Universitario A. Gemelli IRCCS, 00168 Rome, Italy; esther.rossi@policlinicogemelli.it

**Keywords:** thermal ablation, thyroid nodules, laser ablation, immunohistochemistry, differential diagnosis

## Abstract

Thermal ablation (TA) is an increasingly adopted minimally invasive treatment for benign thyroid nodules. However, TA induces marked histological alterations that may simulate thyroid malignancy, creating significant diagnostic pitfalls for pathologists. The present study expands our previous institutional series and further characterizes the morphologic, immunohistochemical, and molecular features of thermally ablated thyroid nodules in order to refine the differential diagnosis with thyroid carcinoma. Fourteen surgically excised thyroid nodules previously treated with laser thermal ablation were retrospectively analyzed. Histopathological evaluation focused on architectural changes, nuclear atypia, capsule alterations, degenerative phenomena, and evidence of invasion. Immunohistochemical analysis included galectin-3 (Gal-3), HBME-1, BRAF V600E, p53, and Ki-67. In addition, molecular profiling for the principal thyroid cancer-related alterations, including BRAF, RAS family genes, TERT promoter mutations, PIK3CA alterations, and RET rearrangements, was performed using targeted next-generation sequencing. All nodules showed treatment-related reactive and degenerative changes, including fibrosis/sclerosis, subcapsular hemorrhage, focal oncocytic metaplasia, and architectural distortion. No true capsular or vascular invasion was identified. Immunohistochemically, all cases were negative for Gal-3 and BRAF V600E, while HBME-1 expression was absent or only focally weak. Ki-67 proliferative activity remained consistently low (<3%) in all cases. Molecular analyses did not identify pathogenic alterations involving BRAF, RAS, TERT promoter, PIK3CA, or RET genes in any case. Thermal ablation induces reproducible reactive and degenerative histologic alterations that may closely mimic follicular or papillary thyroid neoplasms. The absence of malignancy-associated immunohistochemical and molecular alterations strongly supports the benign nature of these lesions and highlights the importance of an integrated morphologic, immunohistochemical, and molecular diagnostic approach in challenging post-ablation specimens. Thermally ablated thyroid nodules may display significant pseudo-neoplastic changes that can lead to overdiagnosis of carcinoma. Awareness of these treatment-related alterations, combined with immunohistochemical and molecular profiling, represents a reliable strategy to distinguish reactive post-ablation changes from true thyroid malignancy and to avoid inappropriate clinical management.

## 1. Introduction

Nodular thyroid disease is a common benign condition with higher prevalence in women, iodine-deficient areas, and following radiation exposure [1,2]. The estimated prevalence widely ranges from 2–6% by palpation, 19–35% by ultrasonography, and up to 8–65% in autopsy series [1]. Most benign thyroid nodules (BTNs) are asymptomatic, but large or compressive lesions may provoke dysphagia, dyspnea, local discomfort, or cosmetic issues, thus requiring clinical intervention [1,2,3]. The diagnostic confirmation is based on ultrasound-guided fine needle aspiration cytology (FNAC), according to the Bethesda system categorization. Treatment options include pharmaceutical suppression, usually not effective; surgical thyroidectomy; and TA methods, namely LA, radiofrequency ablation (RFA), and microwave ablation (MWA) [1,2,3,4].

TA causes controlled coagulative necrosis through hyperthermia, followed by fibrosis, progressive remodeling, and partial reabsorption. Compared with surgery, these procedures may offer better cosmetic results, quicker recovery, and less morbidity, while preserving thyroid function in most appropriately selected patients [2,5,6,7]. Long-term studies confirm volume reductions of 50–80% at 3–5 years, with regrowth rates of 4–20%, often due to incomplete marginal ablation or persistence of viable peripheral tissue [8,9]. Indications include symptomatic solid BTNs (>20 mL), autonomous “hot” nodules not suitable for radioiodine, or palliative approach to low-risk recurrent PTMC [1,10,11]. Recent guidelines from the American Thyroid Association, Korean Society of Thyroid Radiology, and European Thyroid Association support the use of TA for selected benign and low-risk malignant nodules [3,12,13].

Although TA is mainly intended to avoid surgery, a subset of patients may still undergo thyroidectomy during follow-up. Surgery may be required because of persistent compressive or cosmetic symptoms, incomplete volumetric response, regrowth of residual viable tissue, patient preference, or renewed clinical, radiological, or cytological concern. In these cases, the pathologist may evaluate specimens that have undergone substantial post-treatment modification. Surgical excision may therefore be indicated in cases of non-responders or persistent symptoms, where changes attributed to TA, such as fibrosis, subcapsular hemorrhage, nuclear clearing, architectural distortion, and pseudocapsular invasion, can simulate follicular neoplasm or carcinoma, leading to difficult diagnosis [1,14,15,16,17].

The diagnostic challenge is related to the fact that thermal injury does not produce a single uniform histological pattern, but rather a spectrum of degenerative, ischemic, and reparative changes. Artifacts resulting from thermal coagulation include subsequent coagulative necrosis, sclerosis, and metaplastic oncocytic changes [18,19]. Residual benign follicles may become entrapped within fibrotic or hemorrhagic tissue, creating a pseudo-invasive appearance. Similarly, nuclear clearing or follicular crowding may raise concern for papillary thyroid carcinoma-like changes or follicular-patterned neoplasia, particularly when the clinical history of previous ablation is not available to the pathologist.

For this reason, the interpretation of post-TA thyroid specimens requires careful integration of morphology, pre-ablation cytology, imaging findings, and treatment history. Ancillary IHC for Gal-3, HBME-1, BRAF V600E, and p53, along with Ki-67 proliferation indexing, is helpful in differentiating these artifacts from neoplastic progression [1,20,21,22]. In particular, a limited immunohistochemical panel may provide useful reassurance when morphologic alterations are striking but lack unequivocal invasive features.

Our group has previously reported histological findings in seven TA-treated nodules, drawing attention to diagnostic pitfalls and the utility of immunohistochemistry in this setting [1]. The present study expands that initial experience to a total of fourteen cases and, importantly, incorporates molecular profiling in addition to detailed morphological and immunohistochemical assessment. Although surgery after successful thermal ablation is relatively uncommon, thereby limiting the availability of histological material, the present cohort represents one of the largest single-institution pathological series specifically addressing the differential diagnosis between post-ablation reactive changes and thyroid malignancy [14,15,23,24]. By integrating morphological, immunohistochemical, and molecular findings, this study aims to refine the distinction between reactive post-ablation alterations and true thyroid carcinoma, helping to avoid diagnostic overinterpretation and inappropriate clinical management.

## 2. Results and Discussion

The clinical and morphological features are summarized in Table 1.

Morphologically, all nodules showed a variable combination of reactive, degenerative, and reparative changes attributable to previous thermal injury. A fibrous capsule or capsule-like rim was present in nearly all cases. Its appearance varied from thin and regular to thickened, irregular, and focally distorted. In several cases, the capsule was associated with adjacent hemorrhage, fibrosis, or sclerosis, creating areas that could potentially simulate capsular involvement. However, no unequivocal true capsular invasion was identified. Suspicious foci were interpreted as entrapment or displacement of benign-appearing follicles within post-treatment fibrosis or hemorrhagic tissue rather than as genuine neoplastic penetration of the capsule.

The predominant architectural pattern was follicular in most cases, whereas a solid arrangement was observed only in a minority. Focal architectural distortion was frequent, especially close to the presumed ablation zone. In these areas, follicles appeared collapsed, irregularly spaced, or compressed by fibrosis and sclerosis. Occasional microfollicular-like or solid/trabecular areas were identified, but these were limited and lacked infiltrative growth, mitotic activity, tumor-type necrosis, or vascular invasion. These findings supported a post-treatment reparative process rather than a true follicular-patterned neoplasm.

**Figure 1:**
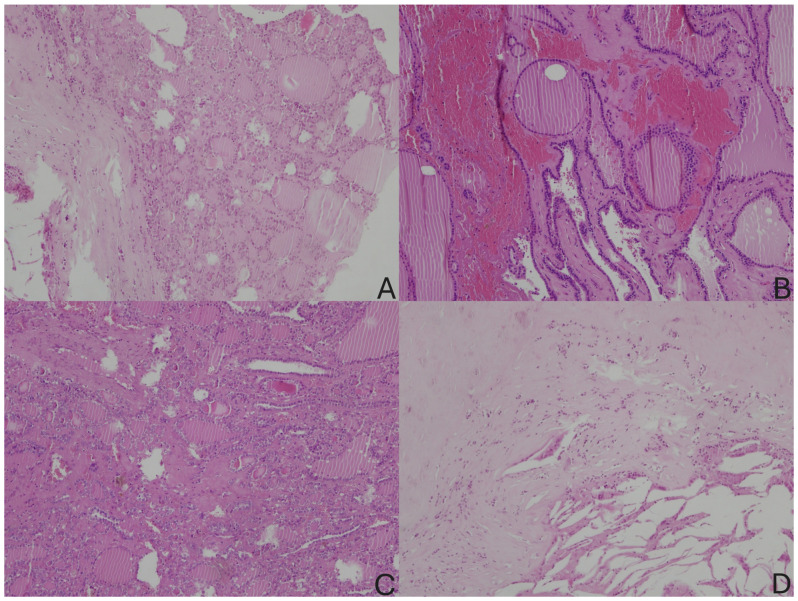
Representative histopathological findings of thermally ablated thyroid nodules: (**A**) Focal oncocytic metaplasia within residual follicles (hematoxylin and eosin, 100×; scale bar = 100 µm). (**B**) Areas of hemorrhage and fibrous capsule with adjacent follicular architecture and no evidence of true capsular invasion (hematoxylin and eosin, 100×; scale bar = 100 µm). (**C**) Focal nuclear clearing in residual follicles adjacent to post-treatment fibrosis (hematoxylin and eosin, 200×; scale bar = 50 µm). (**D**) Sclerosis with occasional multinucleated giant cells and reparative stromal reaction (hematoxylin and eosin, 100×; scale bar = 100 µm).

Nuclear characteristics showed dark, basophilic, and relatively uniform nuclei in most cases, while clear or pale nuclei were observed in a subset of nodules. The latter were sometimes associated with mild enlargement or overlapping, but these changes were focal and topographically related to areas of thermal injury. They were not accompanied by convincing nuclear grooves, pseudoinclusions, or diffuse papillary thyroid carcinoma-type nuclear features. Representative histological findings, including clear nuclei, fibrous capsule with follicular architecture, oncocytic cells, and sclerosis with multinucleated giant cells, are shown in Figure 1.

Degenerative and reparative alterations were common. Subcapsular hemorrhage was present in nearly all cases and was often associated with organization, hemosiderin deposition, or stromal reaction. Sclerosis and fibrosis were also frequent, usually with a zonal distribution and greater intensity near the ablation site. Focal ischemic-type changes with hyalinized vessels were observed in a minority of cases, whereas coagulative necrosis was limited. Importantly, neither true capsular invasion nor vascular invasion was identified in any case.

Oncocytic metaplasia was present in half of the cases. These oncocytic cells showed abundant eosinophilic granular cytoplasm and were generally arranged in residual follicles, often at the periphery of the treated area. The oncocytic change was focal or moderate rather than diffuse and was not associated with destructive invasion, increased proliferative activity, or molecular evidence of neoplasia. Therefore, it was interpreted as a reactive metaplastic phenomenon within the post-ablation spectrum.

**Figure 2:**
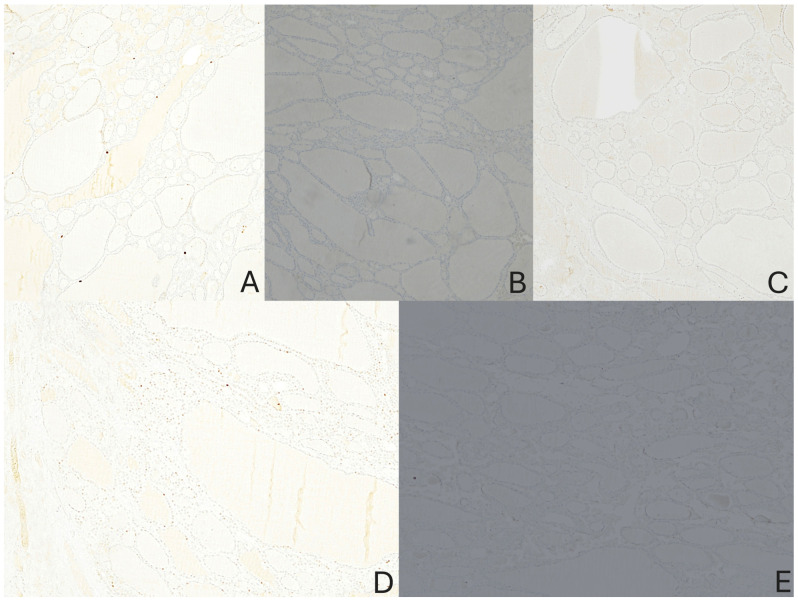
Immunohistochemical findings in laser thermal ablation-treated thyroid nodules: (**A**) Low Ki-67 labeling index, supporting the absence of a significant proliferative component; 100× magnification; scale bar, 100 µm. (**B**) Absence of Galectin-3 expression; 100× magnification; scale bar, 100 µm. (**C**) Absent BRAF V600E expression; 100× magnification; scale bar, 100 µm. (**D**) Focal weak p53 expression, interpreted as stress-related rather than mutation-type overexpression; 100× magnification; scale bar, 100 µm. (**E**) Absent/very weak HBME-1 membranous staining in a limited area adjacent to post-ablation changes; 100× magnification; scale bar, 100 µm.

Immunohistochemical profiling was performed as shown in Table 2 and Figure 2. All cases were negative for Galectin-3 and BRAF V600E. HBME-1 expression was absent in most cases, with only focal and weak membranous positivity observed in a minority of nodules, mainly in microfollicular areas close to the ablation zone. This isolated finding was not associated with invasive growth or additional malignant features. p53 positivity was identified in a minority of cases and was focal and weak rather than diffuse or strong. The Ki-67 labeling index remained consistently low, below 3% in all cases, confirming the absence of a significant proliferative component. Interobserver agreement was excellent, with kappa values of 0.82 for morphology and 0.89 for IHC interpretation.

Molecular analysis did not reveal pathogenic alterations involving BRAF, RAS genes, TERT promoter, PIK3CA, or RET in any analyzed case. No molecular profile suggestive of thyroid neoplasia was identified. This absence of thyroid cancer-related alterations was particularly relevant in nodules showing focal nuclear clearing, architectural distortion, or pseudo-invasive changes, as it reinforced the interpretation that these abnormalities were treatment-related rather than neoplastic.

These findings represent an extension of our previously reported series of seven cases [1], now including molecular analysis and showing slightly increased rates of hemorrhage and a balanced prevalence of oncocytic changes. These differences may reflect the increased sample size and variability in the post-treatment interval after TA, emphasizing the dynamic spectrum of degeneration, remodeling, and repair within ablated thyroid nodules.

The current series of twelve cytologically benign thyroid nodules subjected to laser ablation confirms, refines, and significantly expands our initial seven-case experience [1]. The present findings also demonstrate a near-complete histological overlap with post-ablation changes described across different thermal energy modalities, including radiofrequency, microwave, and HIFU [6,14,15,16,23,24,25,26,27]. Although these techniques differ in energy delivery, procedural parameters, and tissue penetration, the final histological response appears largely shared and is dominated by coagulative injury, hemorrhage, fibrosis, sclerosis, and tissue remodeling.

The current series of fourteen cytologically benign thyroid nodules subjected to laser ablation confirms, refines, and expands our initial seven-case experience [1]. The present findings also demonstrate a near-complete histological overlap with post-ablation changes described across different thermal energy modalities, including radiofrequency, microwave, and high-intensity focused ultrasound [6,14,15,16,23,24,25,26,27]. Although these techniques differ in energy delivery, procedural parameters, and tissue penetration, the final histological response appears largely shared and is dominated by coagulative injury, hemorrhage, fibrosis, sclerosis, and tissue remodeling.

The most consistent and diagnostically impactful changes remain the formation of a well-defined fibrous capsule, prominent subcapsular hemorrhage, and dense zonal fibrosis or sclerosis centered on the ablation site. These findings represent the stereotypical repair response to laser-induced tissue injury and are virtually indistinguishable from those observed following RFA or MWA. Their recognition is essential because these post-treatment alterations may significantly modify the relationship between the nodule, capsule, and adjacent thyroid parenchyma, generating appearances that can be misinterpreted as neoplastic growth.

A major diagnostic issue in post-ablation thyroid specimens is the distinction between true invasion and pseudoinvasion. In conventional thyroid pathology, capsular and vascular invasion are key criteria for the diagnosis of follicular carcinoma and other follicular-patterned malignant neoplasms. However, after TA, the capsule and pericapsular tissue may be distorted by fibrosis, hemorrhage, ischemic change, follicular displacement, and reparative stromal reaction. Residual benign follicles may appear entrapped within fibrotic tissue or close to the capsule, creating a pseudo-invasive appearance. In our series, suspicious foci lacked the defining features of true invasion: there was no complete capsular transgression by a proliferative tumor front, no endothelial-lined vascular space containing tumor thrombi, and no associated infiltrative malignant growth.

Architectural rearrangement toward solid or microfollicular patterns, combined with focal nuclear clearing, may raise concern for an encapsulated follicular neoplasm or follicular variant of papillary thyroid carcinoma. However, several reproducible clues reliably indicate a reactive, post-ablative process rather than true neoplasia: first, the strict zonal distribution of atypical features, with maximum intensity near the ablation center and gradual fading toward the periphery; second, the complete absence of genuine capsular or vascular invasion, despite occasional pseudoinvasion of the fibrous capsule by reactive follicles or hemorrhage; third, the presence of oncocytic metaplasia, typically peripheral and limited, which is a well-recognized reactive phenomenon following thermal injury [1,15,18]; and fourth, negligible mitotic activity and uniformly low Ki-67 labeling index.

Another relevant pitfall is represented by nuclear changes. In our cohort, clear nuclei were observed in a subset of cases, sometimes in association with mild enlargement or overlapping. These features may resemble papillary thyroid carcinoma-like atypia when assessed in isolation. However, in post-ablation nodules, nuclear changes are generally focal, incomplete, and spatially related to areas of tissue injury. They do not show the diffuse and coherent nuclear pattern expected in papillary thyroid carcinoma. This distinction is important because overreliance on isolated nuclear clearing without considering the post-treatment context may lead to overdiagnosis.

Oncocytic metaplasia was also frequent in our series. This finding should be interpreted cautiously because oncocytic change may be encountered in both benign and malignant thyroid lesions. In the post-ablation setting, however, oncocytic metaplasia is best regarded as part of the reparative spectrum when it is focal, peripheral, and associated with fibrosis, sclerosis, or ischemic changes. In our cases, oncocytic cells were not associated with destructive invasion, high proliferative activity, or a malignant immunohistochemical profile, supporting their reactive nature [1,15,18].

Immunohistochemistry provides important diagnostic support in this setting [20,21,22]. Complete negativity for Galectin-3 and BRAF V600E, together with a consistently low Ki-67 labeling index, strongly favors a benign post-ablation process. HBME-1 expression was absent in nearly all cases and was limited to focal and weak membranous staining in a minority of nodules. This finding should be interpreted with caution, as occasional HBME-1 reactivity may be encountered in benign thyroid lesions and in reparative or degenerative conditions. In the absence of additional morphological, immunohistochemical, or molecular evidence of malignancy, isolated focal HBME-1 positivity should not be regarded as sufficient evidence of neoplastic transformation. Accordingly, HBME-1 is best interpreted within an integrated diagnostic framework rather than as a stand-alone marker [15,21,24]. p53 positivity was observed in a minority of cases and was consistently focal and weak. Given the absence of morphologic evidence of malignancy and the lack of pathogenic molecular alterations, this staining pattern is more likely to reflect cellular stress and DNA damage responses induced by thermal injury rather than true TP53-driven neoplastic transformation. This interpretation is also supported by the absence of increased proliferative activity, tumor-type necrosis, destructive growth, or vascular invasion. Therefore, focal p53 expression in post-ablation thyroid nodules should be interpreted cautiously and only in relation to the overall morphological and molecular context [28].

In addition to morphology and immunohistochemistry, molecular profiling may represent a useful adjunct in the differential diagnosis between post-ablation reactive atypia and true thyroid neoplasia. In our series, the absence of canonical thyroid cancer-related molecular alterations, including BRAF, RAS, TERT promoter, PIK3CA, and RET abnormalities, further supported the benign and treatment-related nature of these lesions. Although molecular testing is not routinely required in all post-ablation nodules, it may become particularly valuable in diagnostically challenging cases showing pseudo-invasive growth, nuclear clearing, or microfollicular rearrangement after thermal injury.

Although broader genomic approaches such as whole-transcriptome sequencing or comprehensive genomic profiling may detect additional alterations, our objective was not oncogenic discovery but rather the exclusion of the most clinically relevant molecular events associated with thyroid malignancy. In this context, the absence of canonical driver alterations is diagnostically meaningful and supports the interpretation of these findings as reactive rather than neoplastic.

Such comparisons across thermal ablation techniques are remarkably consistent. Identical degenerative patterns after microwave ablation were documented by both Spiezia et al. [14] and Romanelli et al. [24]. Bernardi et al. [15] underscored pseudoinvasion as a common RFA-related mimic of malignancy. Pacella et al. [26], in the largest published surgical series after laser ablation to date, emphasized that diagnostic errors were eliminated by familiarity with these changes combined with a limited IHC panel. Our current cohort reinforces the notion that the histological response is largely modality-independent, reflecting a shared final common pathway of thermal coagulative necrosis followed by organized repair.

The clinical implications are relevant. Failure to recognize these pseudomalignant changes carries the risk of overdiagnosis of carcinoma, with possible unnecessary completion thyroidectomy, central-neck dissection, and radioiodine therapy, with attendant morbidity. Conversely, correct recognition of treatment-related changes allows appropriate reassurance and avoids overtreatment. True malignant transformation after ablation of cytologically benign nodules remains exceedingly rare. In this context, integration of morphology, immunohistochemistry, and molecular profiling may represent the most reliable strategy to avoid overdiagnosis of carcinoma in thermally ablated thyroid nodules.

From a practical diagnostic perspective, our findings support a stepwise approach. First, the pathologist should assess whether atypical features are zonal and centered on the ablation site or whether they form a diffuse autonomous proliferation. Second, the capsule and vessels should be carefully sampled and evaluated using strict criteria for invasion. Third, immunohistochemical stains should be interpreted as a panel rather than individually. Finally, molecular analysis may be reserved for cases in which morphology and immunohistochemistry remain equivocal. This approach is particularly useful in specimens where the clinical history of ablation is incomplete or where post-treatment changes are especially pronounced.

Study limitations include the retrospective design, the relatively limited sample size, and the exclusive inclusion of laser ablation-treated nodules. However, the modest number of cases reflects the intrinsic difficulty of obtaining surgical specimens after thermal ablation, since the primary objective of these procedures is to avoid surgery. Consequently, opportunities for histopathological examination remain relatively uncommon. A further limitation is the potential selection bias introduced by the inclusion of only surgically excised nodules, which may represent a subset of patients with persistent symptoms, incomplete response, regrowth, patient preference, or renewed clinical/radiological concern and may therefore not fully reflect the broader population of thermally ablated nodules. Additionally, molecular analysis was performed using a targeted panel focused on the most clinically relevant thyroid cancer-associated alterations rather than a comprehensive genomic or transcriptomic approach. Nevertheless, the absence of canonical thyroid cancer-related molecular events remains diagnostically informative in the specific context of post-ablation atypia. Despite these limitations, the remarkable consistency between our findings and those reported across different thermal ablation modalities supports the broader applicability of the observed histopathological patterns [6,14,15,16,23,24,25,26,27,28,29,30].

Overall, laser thermal ablation produces a reproducible constellation of post-treatment changes that may mimic thyroid carcinoma but can be recognized through careful morphological analysis and appropriate ancillary testing. The absence of true invasion, the zonal distribution of atypia, the low proliferative index, the lack of diffuse malignancy-associated immunohistochemical expression, and the absence of canonical thyroid cancer-related molecular alterations all support a benign post-ablative process. Familiarity with these findings is essential to avoid diagnostic overinterpretation and inappropriate clinical management.

## 3. Materials and Methods

Between 2018 and 2025, we retrieved from the pathology archives of the University of Messina and “San Vincenzo” Hospital in Taormina, Italy, a series of fourteen thyroid nodules that had undergone percutaneous laser ablation (LA) before surgical removal. These cases were selected according to strict criteria: all nodules had shown a definitively benign cytological diagnosis on fine-needle aspiration cytology performed before ablation (Bethesda category II); complete clinical, ultrasonographic, and follow-up imaging data had to be available; and the final surgical histology had to confirm the benign nature of the lesion. All cases with a history of thyroid or extrathyroidal malignancies, incomplete documentation, or nodules treated with other thermal ablation techniques, such as radiofrequency or microwave ablation, were excluded.

The patient cohort consisted of 14 individuals with a mean age of 56 years, reflecting a relatively mature demographic typical for thyroid nodule presentations. There was a clear female predominance, with women comprising 71.4% of the group and men accounting for the remaining 28.6%. The nodules were solitary in the majority of cases, whereas multinodular configurations were observed in one-third of the cohort. The mean pre-TA volume was 27.8 mL, with a range of 18–44 mL. The post-TA surgical interval ranged from 6 to 18 months, with a mean of 10.7 months, allowing the assessment of both relatively early and more organized post-treatment changes.

During the available follow-up period, no patient developed histological evidence of thyroid malignancy, local recurrence, or disease progression. No additional thyroid-specific treatment was required after surgery.

The reasons for surgery after thermal ablation were recorded whenever available and included persistent compressive or cosmetic symptoms, incomplete volumetric response, regrowth of residual viable tissue, patient preference, or renewed clinical/radiological concern. The interval between ablation and surgery was also recorded for each case.

All hematoxylin-eosin-stained slides available from the resection specimens were independently reviewed by three experienced pathologists who were blinded to the clinical indication for surgery, radiological follow-up findings, immunohistochemical results, and molecular results. Review was specifically directed at histological parameters potentially modified by thermal injury, including predominant architectural pattern, nuclear appearance, capsule integrity, presence and extension of degenerative phenomena, coagulative necrosis, fibrosis/sclerosis, hemorrhage, ischemic changes, signs of capsular or vascular invasion, and oncocytic metaplasia. Histological evaluation was performed using a Nikon Eclipse Ci-L microscope (Nikon Corporation, Tokyo, Japan). Representative photomicrographs were acquired using the NIS-Elements F imaging software v5.42.07 (Nikon Corporation, Tokyo, Japan).

Capsular and vascular invasion were assessed according to current WHO diagnostic criteria for thyroid tumors. Capsular invasion was defined as complete transgression of the capsule by a proliferative tumor front, whereas vascular invasion required the presence of tumor thrombus within an endothelial-lined vascular space, preferably attached to the vessel wall. Foci of benign follicles entrapped within fibrosis, hemorrhage, or reparative tissue were interpreted as pseudoinvasion when these strict criteria were not fulfilled.

Immunohistochemical studies were performed on 4 µm thick sections of formalin-fixed, paraffin-embedded tissue to further characterize the ablated nodules and search for possible subtle signs of malignancy or proliferative activity masked by thermal effects. On a Ventana Benchmark automated platform, staining was performed with the following panel of antibodies: Galectin-3 (Gal-3, rabbit polyclonal, 1:200, overnight incubation; Ylem, Rome, Italy), HBME-1 (mouse monoclonal, 1:100; Dako, Glostrup, Denmark), BRAF V600E mutation-specific antibody (mouse monoclonal, VE1 clone, 1:100; ThermoFisher Scientific, Waltham, MA, USA), p53 (mouse monoclonal, DO-7 clone, 1:50; ThermoFisher Scientific, Waltham, MA, USA)), and Ki-67 (mouse monoclonal, MIB-1 clone, 1:200; ThermoFisher Scientific, Waltham, MA, USA). Heat-induced epitope retrieval was done in EDTA buffer at pH 8.0 for 30 min. Immunoreactivity was semi-quantitatively categorized as negative (<5% of cells), focal/weak (5–20%), or diffuse/strong (>20%). Immunoreactivity for Galectin-3, HBME-1 and BRAF V600E was categorized as negative (<5%), focal/weak (5–20%), or diffuse/strong (>20%). p53 expression was recorded as negative, focal/weak, or diffuse/strong nuclear staining. Ki-67 was reported as the percentage of positive nuclei. p53 staining was interpreted according to staining extent and intensity, with focal weak positivity considered distinct from diffuse strong overexpression. The proliferative fraction was also estimated more precisely by calculating the Ki-67 labeling index in ten randomly selected high-power fields with the aid of digital image analysis software (Aperio ScanScope v12.4.6).

Molecular analyses were performed on representative formalin-fixed paraffin-embedded (FFPE) tissue sections obtained from thermally ablated thyroid nodules. After DNA extraction using the QIAcube system (Qiagen, Hilden, Germany) and the AllPrep DNA/RNA FFPE Kit (Qiagen), molecular profiling was carried out to investigate the presence of the most common genetic alterations associated with thyroid neoplasia.

Targeted mutational analysis included BRAF (including BRAF V600E), RAS family genes (NRAS, HRAS, KRAS), TERT promoter mutations, PIK3CA alterations, and RET rearrangements. Molecular testing was performed using a targeted next-generation sequencing (NGS) approach based on the Myriapod NGS Cancer Panel DNA platform (Diatech Pharmacogenetics, Jesi, Italy), according to the manufacturer’s instructions, as previously described [25]. Sequencing was performed with a mean coverage depth exceeding 2000×. Variants were considered for analysis when supported by a minimum variant allele frequency (VAF) of 5% and adequate read quality metrics.

Correlation analyses were performed between molecular findings and histologic/immunohistochemical features to assess whether thermally induced atypia was associated with underlying neoplastic molecular signatures.

Interobserver reproducibility for the main morphological features was determined using Cohen’s kappa coefficient. Calculated values >0.80 were considered indicative of excellent agreement.

The study was conducted in accordance with the Declaration of Helsinki and approved by the institutional ethics committee, under Protocol No. 65-22 July 2022. A waiver of informed consent was provided in light of the retrospective design and use of fully anonymized archival material.

## 4. Conclusions

Laser thermal ablation of benign thyroid nodules causes distinctive and reproducible histological changes, including dense fibrosis, subcapsular hemorrhage, pseudocapsular invasion, architectural distortion, and oncocytic metaplasia, which can mimic malignancy. The combination of judicious morphological assessment with a limited immunohistochemical panel, including Galectin-3, HBME-1, BRAF V600E, p53, and Ki-67, represents a dependable strategy to distinguish treatment-related changes from true neoplastic transformation. Molecular profiling may provide additional reassurance in diagnostically challenging cases by excluding canonical thyroid cancer-related alterations. Awareness of these reproducible post-ablation alterations is increasingly important as thermal ablation becomes more widely adopted in clinical practice. Recognition of their characteristic morphological, immunohistochemical, and molecular profile may prevent unnecessary diagnoses of carcinoma and the consequent risk of overtreatment.

## Figures and Tables

**Table 1:** Patients’ characteristics and nodules’ morphological features (N = 14).

Patients’ Features
Age, mean 56 yrs
Gender n (%)
*Male 4 (28.4%)*
*Female 10 (71.6%)*
Architecture n (%)
*Follicular 12 (85.7%)*
*Solid 2 (14.3%)*
Nuclei n (%)
*Dark 10 (71.6%)*
*Clear 4 (28.4%)*
Number of nodules n (%)
*Single nodule*
*Plurinodular*
Capsule n (%)
*Yes 13 (92.8%)*
*No 1 (7.2%)*
Necrosis n (%)
*Yes 1 (7.2%)*
*No 13 (92.8%)*
Fibrosis n (%)
*Yes 10 (71.6%)*
*No 4 (28.4%)*
Hemorrhage n (%)
*Yes 11 (78.6%)*
*No 3 (21.4%)*
Ischemia n (%)
*Yes 2 (14.3%)*
*No 12 (85.7%)*
Capsular invasion n (%)
*Yes 0 (0%)*
*No 14 (100%)*
Vascular invasion n (%)
*Yes 0 (0%)*
*No 14 (100%)*
Presence of oncocytic cells n (%)
*Yes 7 (50%)*
*No 7 (50%)*

**Table 2:** Immunohistochemical and molecular profile (N = 14).

Markers	Results	Total n.14 (%)
Galectin-3	Positive	0
	Negative	14 (100%)
HBME-1	Positive	1 (7.1%)
	Negative	13 (92.9%)
p53	Positive	3 (21.4%)
	Negative	11 (78.6%)
BRAF-V600E	Positive	0
	Negative	14 (100%)
MIB-1 (Ki-67)	<3%	14 (100%)
Molecular alterations	0%	14 (100%)

## Data Availability

The original contributions presented in this study are included in the article. Further inquiries can be directed to the corresponding authors.
